# Bone quality makes a difference

**DOI:** 10.1080/17453674.2021.1941632

**Published:** 2021-06-28

**Authors:** Hannu T. Aro

**Affiliations:** Department of Orthopaedic Surgery and Traumatology, Turku University Hospital and University of Turku, Turku, Finland

In total hip arthroplasty, poor bone quality is a major risk factor for complications. There is increasing evidence that osteoporotic patients and particularly osteoporotic females should receive a cemented prosthesis to avoid peroperative or early postoperative periprosthetic fractures, and reduce the risk of poor fixation resulting in pronounced subsidence eventually resulting in clinical loosening.

In cementless total hip arthroplasty, adequate initial femoral stem stability is necessary for clinical success. Clinical subsidence, measurable from routine radiographs, predisposes femoral stems to early failure (Warth et al. 2020). Avoiding undersizing the femoral stem with a meticulous broaching technique may minimize the risk of subsidence (Warth et al. 2020). The acceptable level of subsidence of cementless stems, as determined by radiostereometric analysis, is still unknown (van der Voort et al. 2015). Minimal subsidence (mean 0.8 mm) has resulted in faster recovery of walking speed and walking activity with improved patient-reported outcomes (Aro et al. 2021).

In this issue of Acta Orthopaedica, Dyreborg et al. (2021) present results from a secondary analysis of clinical trial data. Based on dual-energy X-ray absorptiometry (DXA), the cohort (age ≤ 75 years) involved subjects with normal or osteopenic bone mineral density (BMD) of the femoral neck. The authors found no relationship between hip BMD and postoperative femoral stem subsidence.

In line with the results of Dyreborg et al. (2021), total hip BMD measured by DXA failed to identify women prone to stem subsidence (Nazari-Farsani et al. 2020). DXA seems to overestimate BMD of osteoarthritic hips, explaining its ability to predict stem migration. Naturally, DXA measurements from other sites (contralateral hip, lumbar spine, and distal radius) are important in the detection of undiagnosed osteoporosis.

Interestingly, peripheral DXA evaluation of bone quality seems to work better than hip DXA. BMD of the distal radius, measured by DXA, and cortical-bone thickness of the distal radius, measured by pulse-echo ultrasonometry, may help discriminate women at high risk of stem subsidence (Nazari-Farsani et al. 2020). Explaining the risk of subsidence, women with low cortical-bone thickness of the distal radius had lower total hip BMD and reduced thickness of the medial cortex of the proximal femur with lower stem-to-canal fill ratios.

Cementless stems differ in the means of obtaining cortical contact and initial stability (Khanuja et al. 2011). Stem design influences the risk of periprosthetic femoral fracture (Thien et al. 2014), postoperative bone remodeling (Karachalios et al. 2019), and stem subsidence (de Vries et al. 2014). Parallel-sided femoral stems are designed to engage the metaphyseal cortical bone in the mediolateral plane only. The stem type requires adequate bone stock and unaltered femoral geometry (Grayson and Meneghini 2017). This important instructional notice raises questions. What are the criteria for normal bone stock? Is DXA-measured osteopenia a contraindication?

A parallel-sided femoral stem showed subsidence (mean 1.8 mm) even in postmenopausal women with normal hip BMD and Dorr A/B femur anatomy (Nazari-Farsani et al. 2021). Analyses with quantitative computed tomography (QCT) revealed that the stem subsidence occurred in women with high bone turnover and decreased volumetric BMD of the intertrochanteric region (Aro et al. 2021). This region is critical for the stem stability.

In ageing women, endosteal trabeculation and increased intracortical porosity of the proximal femur (Zebaze et al. 2010) pose natural difficulties in achieving stem stability. In men, factors causing stem subsidence seem to be different, including young age, high bodyweight, and increased early postoperative activity (Bottner et al. 2005).

Even in women, physical activity seems to dictate the direction of stem rotation. Femoral stems are affected by high torsional moments during daily activities (Bergmann et al. 2001) and postoperative walking activity, aside from total hip BMD, is associated with stem rotation in postmenopausal women (Nazari-Farsani et al. 2021). Early walking activity creates the typical pattern of slight internal stem rotation, while stems do not rotate in women with low walking activity.

Stem migration does not seem to be a random event. DXA is insensitive to critical, albeit subtle, structural changes of the proximal femur. In the future, emerging robotic techniques may allow routine measurement of local volumetric BMD by means of preoperative QCT. Such an approach could improve screening of bone quality and help patient selection for the use of cementless techniques.

## Potential conflicts of interest

The author has received a consultation fee from UCB Biopharma Sprl (Belgium) and institutional research grants from the Academy of Finland and Amgen Inc. (USA).


Hannu T. Aro *Department of Orthopaedic Surgery and Traumatology, Turku University Hospital and University of Turku, Turku, Finland*
*Email:*
hannu.aro@utu.fi


